# Efficacy and Safety of Concurrent Chemoradiotherapy Combined With Induction Chemotherapy or Adjuvant Chemotherapy in Patients With Stage II–IVA Nasopharyngeal Carcinoma: A Propensity Score Matching Analysis and Meta-Analysis

**DOI:** 10.3389/fonc.2021.778836

**Published:** 2021-12-03

**Authors:** Jie Yang, Zhong-Guo Liang, Yu-Ting Jiang, Kai-Hua Chen, Ling Li, Song Qu, Xiao-Dong Zhu

**Affiliations:** ^1^ Department of Radiation Oncology, Guangxi Medical University Cancer Hospital, Nanning, China; ^2^ Department of Oncology, Wuming Hospital of Guangxi Medical University, Nanning, China

**Keywords:** nasopharyngeal carcinoma, induction chemotherapy (IC), adjuvant chemotherapy (AC), concurrent chemoradiotherapy (CCRT), propensity score-matched analysis, meta-analysis

## Abstract

**Purpose:**

To evaluate the efficacy and safety of induction chemotherapy (IC) combined with concurrent chemoradiotherapy (CCRT) *versus* CCRT combined with adjuvant chemotherapy (AC) in patients with stage II–IVA nasopharyngeal carcinoma (NPC), we conducted a retrospective study and a meta-analysis combining the results of our studies.

**Patients and Methods:**

We used the propensity score matching (PSM) to balance variables. A total of 168 patients were chosen by one-to-two PSM, including 101 patients with IC + CCRT and 67 cases with CCRT + AC. We used the Kaplan–Meier curve to compare survival outcomes and also used Cox regression analysis to determine independent prognostic factors. For meta-analysis, we determined the related studies by searching the PubMed database. We used STATA v12 software to perform meta-analysis of the extracted data and calculate pooled hazard ratios, 95% confidence intervals of survival outcomes, and risk ratios for the toxicities.

**Results:**

In this retrospective study, there was no significant difference in 5-year overall survival (76.9% *vs*. 79.0%, *P* = 0.966), progression-free survival (71.3% *vs*. 68.5%, *P* = 0.332), distant metastasis-free survival (80.5% *vs*. 74.2%, *P* = 0.140), and locoregional relapse-free survival (91.5% *vs*. 91.8%, *P* = 0.894) among patients with NPC with IC + CCRT *versus* CCRT + AC after PSM. For meta-analysis, six articles (including our study) reporting 1,052 cases of IC + CCRT and 883 cases of CCRT + AC were included in the meta-analysis. There was no difference of OS (pooled HR = 0.90, 95% CI: 0.63–1.29, *P* = 0.561), PFS (pooled HR = 1.07, 95% CI: 0.87–1.33, *P* = 0.633), DMFS (pooled HR= 0.98, 95% CI: 0.76-1.25, *P*=0.861), and LRRFS (pooled HR = 1.06, 95% CI: 0.76–1.48, *P* = 0.724).

**Conclusion:**

The efficacy of IC + CCRT and CCRT + AC was comparable in patients with stage II–IVA NPC. In terms of compliance and acute adverse reactions, IC + CCRT may be a potential therapeutic strategy.

## 1 Introduction

Nasopharyngeal carcinoma (NPC) is a malignant tumor of the head and neck with a distinct geographic distribution. It was estimated that there were 129,079 new cases of NPC worldwide in 2018, while 72,987 patients would die from NPC (1). NPC is closely associated with Epstein–Barr virus (EBV) infection, and some studies have shown (2, 3) that EBV DNA testing of plasma specimens is important for the screening of early asymptomatic NPC. Due to the special location of the nasopharynx and the radiosensitive behavior of NPC cells, radiotherapy has become its main treatment. There is no doubt that the rate of local control of advanced NPC has improved with the advent of intensity-modulated radiotherapy (IMRT). Despite the use of concurrent chemotherapy, distant metastasis and recurrence remain its main mode of treatment failure (4), occurring in 18% to 27% of patients (5). The Intergroup 0099 Study (6) first established the role and place of chemotherapy in locoregionally advanced NPC (LA-NPC). The addition of chemotherapy, such as induction chemotherapy (IC) and adjuvant chemotherapy (AC), to radiotherapy (RT) may be able to reduce treatment failure due to distant metastases (7). Although there are a few research studies and trials comparing the efficacy and safety between IC + CCRT and CCRT + AC, there is still a lack of large-scale clinical studies comparing the survival and prognosis of the two treatment modalities.

The optimal treatment of IC + CCRT and CCRT + AC in non-metastatic NPC patients is not yet clear. Therefore, we conducted a retrospective study and meta-analysis to investigate the survival and prognosis with stage II–IVA NPC patients of the two treatment modalities. To avoid the interference of the variables of covariates in the two groups, we used propensity score matching (PSM) to balance the variables.

## 2 Material and Methods

### 2.1 Retrospective Study

#### 2.1.1 Patients

Our study integrated data from the Guangxi Medical University Cancer Hospital, which was a retrospective study. Patients who were previously untreated, had histologically confirmed diagnosis of NPC, received IC + CCRT or AC + CCRT, were 18–70 years old, and had stage II–IVA NPC [the eighth edition of the Union for International Cancer Control (UICC)/American Joint Committee on Cancer (AJCC) staging system] were recruited from November 2011 to December 2015. The other inclusion criteria included Karnofsky scale ≥70, no proof of distant metastases, and IMRT as radiotherapy modality, with complete clinical data and follow-up data. Based on these criteria, a total of 362 patients with IC + CCRT (*n* = 192) and CCRT + AC (*n* = 72) were finally included in this study.

#### 2.1.2 Radiotherapy

In this study, all patients received IMRT. The target volumes were designated in accordance with the International Commission on Radiation Units and Measurements reports 50 and 62 (8). The prescribed dose was 70.06–73.92 Gy to the planning target volume (PTV) of the primary gross tumor volume (PGTVnx) in 31–33 fractions, 65.10–72.32 Gy to the PTV of the nodal gross tumor volume (PGTVnd) in 31–32 fractions, 60–62 Gy to the PTV of the first clinical tumor volume (PCTV1, the high-risk target area) in 30–31 fractions, and 54–55.8 Gy to the PTV of the second CTV (PCTV2, the low-risk target area) in 30–31 fractions. All patients received radiotherapy once a day for 5 days per week.

#### 2.1.3 Chemotherapy

During the study period, all patients received chemotherapy and platinum-based agents through IC and AC chemotherapy regimens. The regimens of IC included docetaxel, cisplatin, and 5-florouracil (TPF; 60 mg/m^2^ on day 1, 60 mg/m^2^ on day 1, and 600 mg/m^2^/day on days 1–5, respectively); PF (80 mg/m^2^ on day 1 and 600 mg/m^2^/day on days 1–5, respectively); TP (80 mg/m^2^ on day 1 and 80 mg/m^2^ on day 1, respectively); and gemcitabine and cisplatin (GP; 1,000 mg/m^2^ on day 1 and 80 mg/m^2^ on day 1, respectively). The regimens of AC included TPF, PF, TP, and GP, the doses of which were consistent with the IC regimen. IC and AC regimens were repeated every 3 weeks.

#### 2.1.4 Follow-Up

After completing treatment, all patients were followed up every 30–90 days during the first 2 years, every 180 days for the next 3 years, and every 1 year thereafter until death. To assess the disease status and treatment toxicity of the patients, during the follow-up periods, physical examination, abdominal ultrasonography, chest radiography, and head/neck magnetic resonance imaging (MRI) scans were performed. Cervical, chest, and abdomen plain scan and enhanced computed tomography (CT) were examined when necessary. Toxicity assessment was based on the Common Terminology Criteria for Adverse Events (CTCAE) 4.03 and Radiation Therapy Oncology Group (RTOG) radiation morbidity scoring criteria. Follow-up time was calculated from the date of diagnosis to the date of death or the most recent follow-up or the date of relapse. Overall survival (OS) was defined as the time from the date of diagnosis to the last follow-up visit or to death from any cause. Progression-free survival (PFS) was defined as the time from the date of diagnosis to disease progression (including recurrence, metastasis, and death). Distant metastasis-free survival (DMFS) was defined as the time from the date of diagnosis to distant metastasis, and locoregional relapse-free survival (LRRFS) was defined as the time from the date of local or regional recurrence. Any toxicities and survival data for all patients were recorded in the outpatient and inpatient medical record systems.

#### 2.1.5 Statistical Analysis

Our study was a retrospective study describing the clinical characteristics of the two groups of patients, using the *χ* ^2^ test for categorical variables and the *t*-test for continuous variables to compare the differences in the clinical baseline characteristics of patients in IC + CCRT and CCRT + AC. PSM was used to balance potential prognostic factors. Moreover, a 1:2 matching protocol was used with R (version 3.6.1), and the caliper width was equal to 0.1 of the logit standard deviation of the propensity score. Survival curves were generated using the Kaplan–Meier method and the difference was compared by the log-rank test. In addition, we performed multivariate analysis using the Cox proportional hazards model to determine the significant prognostic factors. Additionally, we calculated hazard ratios (HRs), 95% confidence intervals (CIs), and *P*-values for each independent prognostic factor. All data of this study were analyzed using the program Statistical Package for Social Sciences (SPSS) version 22.0, and *P <*0.05 was considered significant.

### 2.2 Meta-Analysis

#### 2.2.1 Search Strategy

We searched PubMed for all studies comparing IC + CCRT *versus* CCRT + AC in patients with NPC. For this, the following equation was used: “(‘Nasopharyngeal Carcinoma’ OR ‘Carcinoma, Nasopharyngeal’ OR ‘Carcinomas, Nasopharyngeal’ OR ‘Nasopharyngeal Neoplasms’) AND (‘Induction chemotherapy’ OR ‘Induction Chemotherapies’ OR ‘Chemotherapies, Induction’ OR ‘Chemotherapy, Induction’ OR ‘neoadjuvant chemotherapy’) AND (‘Adjuvant chemotherapy’ OR ‘Drug Therapy, Adjuvant’ OR ‘Adjuvant Drug Therapy’).”. The final search date was June 1, 2021.

In total, 42 extracted studies were included that met the following criteria: 1) study type: all prospective clinical trials or retrospective studies comparing the efficacy and/or safety of IC + CCRT *versus* CCRT + AC in patients with NPC; 2) study subjects: all study subjects had a histologically or cytologically confirmed diagnosis of NPC, with no restriction on pathological staging; 3) study treatment patterns: all patients were treated with IC + CCRT or CCRT + AC, and radiotherapy was IMRT. According of the inclusion criteria, a total of six articles (9–13) were finally included, including our study. For the included studies, we extracted information from the studies including the following: first author, year of publication, country, study design, number of cases, time of included cases, follow-up time, staging, and treatment regimen. Survival data (mainly extracted for HRs and its 95% CIs) and adverse events were obtained directly from the included studies. For studies with only survival curves, we prioritized contacting the authors of the original article to see if the HRs and its 95% CIs were available, and if not available, the solution provided by the literature of Tierney et al. (14) could be used by applying the Engauge Digitizer software to extract multiple points on the survival curve, after which the survival rates of the two groups at different follow-up times were derived, and then the data were entered into the Excel sheet provided in the literature of Tierney et al. (14), and the HR values and 95% CIs can be derived from the results page of the Excel sheet.

#### 2.2.2 Quality Assessment and Statistical Analysis

The Newcastle-Ottawa Scale (NOS) was used to appraise the quality of the included retrospective studies in the meta-analysis. Each retrospective study quality score was in the range of 0~9, and a score of 6 or more indicated high-quality studies. Statistical analyses were performed using STATA v12. All survival outcome data (OS, PFS, DMFS, LRRFS) were expressed as HRs and 95% CIs. Risk ratios (RRs) with 95% CIs were used as summary statistics for toxicities. If *P <*0.05 and the 95% CIs did not include the value 1, the estimate of the survival outcomes was considered statistically significant. The observed HRs or RRs <1 indicated survival benefit or less persistent toxicity in patients who were treated with IC + CCRT. Statistical heterogeneity across studies was signified by using the Cochrane *Q* test and the *I* ^2^ statistic (15). Heterogeneity was defined as when the *P*-value was <0.10 of the Cochrane *Q* test or the *I* ^2^ value was >50%. If *P >*0.10 and *I* ^2^ <50%, a fixed-effects model was applied for analysis. If the heterogeneity was small, the random-effects model was used.

## 3 Results

### 3.1 Retrospective Study

#### 3.1.1 Patient Characteristics

From November 2011 to December 2015, we identified 364 patients with NPC receiving either IC + CCRT or CCRT + AC. Among these patients, 292 (80.2%) received IC + CCRT and 72 (19.8%) received CCRT + AC at Guangxi Medical University Cancer Hospital. For the original data, the male (*n* = 272) to female (*n* = 92) ratio was 3.0:1, and the median age was 45 (range 18–70) years old. Before PSM, there were significant differences in baseline characteristics between the IC + CCRT and CCRT + AC in terms of serum lactate dehydrogenase (LDH), concurrent chemotherapy cycles, and IC/AC cycles. After a 1:2 propensity matching score (caliper value 0.1), 168 patients were finally included, of whom 101 were treated with IC + CCRT and 67 with CCRT + AC. **Table 1** shows the baseline characteristics of patients between IC + CCRT and CCRT + AC. No significant differences in potential prognostic factors were observed for IC + CCRT and CCRT + AC after matching.

**Table 1:** Baseline patient characteristics before and after PSM.

Characteristics	Before PSM			After PSM		
	IC + CCRT (*n* = 292)	CCRT + AC (*n* = 72)	*P*-value	IC + CCRT (*n* = 101)	CCRT + AC (*n* = 67)	*P*-value
Gender			0.717			0.678
Male	217 (74.3%)	55 (76.4%)		74 (73.3%)	51 (76.1%)	
Female	75 (25.7%)	17 (23.6%)		27 (26.7%)	16 (23.9%)	
Age at diagnosis (years)			0.956			0.839
18–44	140 (47.9%)	35 (48.6%)		51 (50.5%)	33 (49.3%)	
45–60	133 (45.5%)	33 (45.8%)		46 (45.5%)	30 (44.8%)	
>60	19 (6.5%)	4 (5.6%)		4 (4.0%)	4 (6.0%)	
KPS			0.343			0.294
≥90	273 (93.5%)	65 (90.3%)		96 (95.0%)	60 (89.6%)	
<90	19 (6.5%)	7 (9.7%)		5 (5.0%)	7 (10.4%)	
Histological type (WHO)			0.125			0.260
I–II	50 (17.1%)	18 (25.0%)		17 (16.8%)	16 (23.9%)	
III	242 (82.9%)	54 (75.0%)		84 (83.2%)	51 (76.1%)	
T category			0.803			0.660
T1	5 (1.7%)	2 (2.8%)		1 (1.0%)	2 (3.0%)	
T2	84 (28.8%)	19 (26.4%)		31 (30.7%)	17 (25.4%)	
T3	104 (35.6%)	29 (40.3%)		37 (36.6%)	28 (41.8%)	
T4	99 (33.9%)	22 (30.6%)		32 (31.7%)	20 (29.9%)	
N category			0.101			0.639
N0–1	106 (36.3%)	36 (50%)		41 (40.6%)	32 (47.8%)	
N2	142 (48.6%)	28 (38.9%)		45 (44.6%)	27 (40.3%)	
N3	44 (15.1%)	8 (11.1%)		15 (14.9%)	8 (11.9%)	
Stage^a^			0.287			0.502
II–III	154 (52.7%)	43 (59.7%)		55 (54.5%)	40 (59.7%)	
IV	138 (47.3%)	29 (40.3%)		46 (45.5%)	27 (40.3%)	
HGB, g/L			1.000			1.000
≥120/110 (M/F)	274 (93.8%)	67 (93.1%)		95 (94.1%)	63 (94.0%)	
<120/110 (M/F)	18 (6.2%)	5 (6.9%)		6 (5.9%)	4 (6.0%)	
PLT			0.800			0.517
300 ≤ PLT ≤ 100	179 (61.3%)	44 (61.1%)		60 (59.4%)	43 (64.2%)	
<100	1 (0.3%)	0		1 (1.0%)	0	
>300	112 (38.4%)	28 (38.9%)		40 (39.6%)	24 (35.8%)	
ALB, g/L			0.353			0.151
≥35	285 (97.6%)	72 (100%)		97 (96.0%)	67 (100%)	
<35	7(2.4%)	0		4(4%)	0	
LDH			0.025			0.162
Median	184	168		184	168	
Range	109–759	104–373		109–384	116– 373	
IC/AC cycles			<0.001			0.177
1	20 (6.8%)	21 (29.2%)		17 (16.8%)	17 (25.4%)	
≥2	272 (93.2%)	51 (70.8%)		84 (83.2%)	50 (74.6%)	
CC cycles			<0.001			0.654
1	33 (11.3%)	3 (4.2%)		8 (7.9%)	3 (4.5%)	
2	185 (63.4%)	17 (23.6%)		23 (22.8%)	17 (25.4%)	
≥3	74 (25.3%)	52 (72.2%)		70 (69.3%)	47 (70.1%)	
IC/AC regimen			–			–
TPF	248 (84.9%)	13 (18.1%)		76 (75.2%)	12 (17.9%)	
TP	15 (5.1%)	8 (11.1%)		8 (7.9%)	8 (11.9%)	
PF	26 (8.9%)	51 (70.8%)		16 (15.8%)	47 (70.1%)	
GP	3 (1.0%)	0		1 (1.0%)	0	

PSM, propensity score matching; IC, induction chemotherapy; CCRT, concurrent chemoradiotherapy; AC, adjuvant chemotherapy; KPS, Karnofsky scores; WHO, World Health Organization; HGB, hemoglobin; M/F, male/female; PLT, platelet; ALB, serum albumin; LDH, serum lactate dehydrogenase; alP, serum alkaline phosphatase; sf, serum ferritin; CC, concurrent chemotherapy; tpf, docetaxel, cisplatin, and 5-florouracil; tp, docetaxel and cisplatin; pf, cisplatin and 5-florouracil; gp, gemcitabine and cisplatin.

a According to the eighth edition of the Union for International Cancer Control/American Joint Committee on Cancer (UICC/AJCC) staging system.

#### 3.1.2 Comparisons of Survival Outcomes Before and After PSM

**Figure 1:**
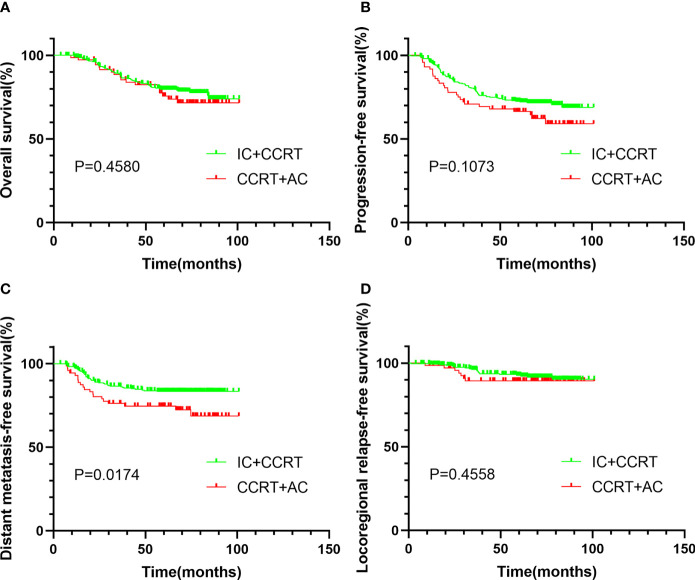
Kaplan–Meier survival curves of OS **(A)**, PFS **(B)**, DMFS **(C)**, and LRRFS **(D)** for patients stratified as IC + CCRT and CCRT + AC before PSM. OS, overall survival; PFS, progression-free survival; DMFS, distant metastasis-free survival; LRRFS, locoregional relapse-free survival; IC, induction chemotherapy; AC, adjuvant chemotherapy; CCRT, concurrent chemoradiotherapy; PSM, propensity score matching.

The median follow-up time before matching was 65.8 months (range 3.8–100.9 months). The 5-year OS, 5-year PFS, 5-year DMFS, and 5-year LRRFS of 364 patients were 78.8%, 71.3%, 81.6%, and 92.0%, respectively. Compared with CCRT + AC, IC + CCRT has no significant difference in the 5-year OS (79.6% *vs*. 75.7%, *P* = 0.458, **Figure 1A**), 5-year PFS (72.5% *vs*. 66.4%, *P* = 0.107, **Figure 1B**), and 5-year LRRFS (92.7% *vs*. 89.3%, *P* = 0.456, **Figure 1D**), but it is statistically significant in DMFS (83.4% *vs*. 74.6%, *P* = 0.017, **Figure 1C**).

**Figure 2:**
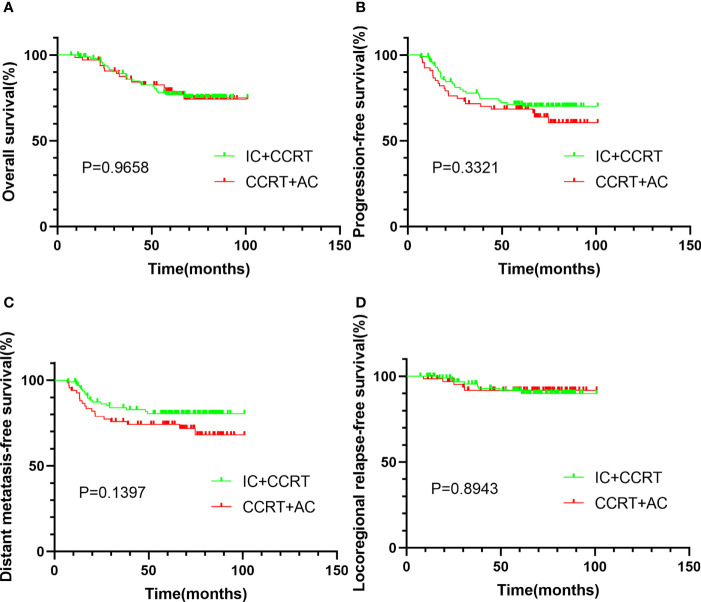
Kaplan–Meier survival curves of OS **(A)**, PFS **(B)**, DMFS **(C)**, and LRRFS **(D)** for patients stratified as IC + CCRT and CCRT + AC after PSM. OS, overall survival; PFS, progression-free survival; DMFS, distant metastasis-free survival; LRRFS, locoregional relapse-free survival; IC, induction chemotherapy; AC, adjuvant chemotherapy; CCRT, concurrent chemoradiotherapy; PSM, propensity score matching.

After PSM, a total of 168 patients were enrolled. The median follow-up time was 65.2 months (range 7.3–100.9 months). After matching, the 5-year OS, 5-year PFS, 5-year DMFS, and 5-year LRRFS were 77.7%, 70.2%, 77.9%, and 91.7%, respectively. Between IC + CCRT and CCRT + AC, the 5-year OS (76.9% *vs*. 79.0%, *P* = 0.966, **Figure 2A**), 5-year PFS (71.3% *vs*. 68.5%, *P* = 0.332, **Figure 2B**), 5-year DMFS (80.5% *vs*. 74.2%, *P* = 0.140, **Figure 2C**), and 5-LRRFS (91.5% *vs*. 91.8%, *P* = 0.894, **Figure 2D**) showed no significant difference.

#### 3.1.3 Univariate and Multivariate Cox Regression Analysis

We used univariate analysis (**Table 2**) and multivariate analysis (**Table 3**) to assess prognostic factors. In the multivariate analysis of our study, the variables included were those with *P <*0.05 in the univariate analysis and treatment method (IC + CCRT *vs*. CCRT + AC). In the multivariate analysis of all 167 patients after PSM, gender (male *vs*. female) and age (18–44 *vs*. >60 years) were independent prognostic factors for OS, PFS, and DMFS. Stage (II-III *vs*. IVA) was the independent prognostic factor for OS (HR = 2.41, 95% CI: 1.23–4.67, *P* = 0.009), PFS (HR = 2.77, 95% CI: 1.57–4.91, *P* < 0.001), DMFS (HR = 2.49, 95% CI: 1.27–4.88, *P* = 0.008) and LRRFS (HR = 3.59, 95% CI: 1.10–11.72, *P* = 0.034). Treatment method was not the independent prognostic factors for OS, PFS, DMFS, and LRRFS.

**Table 2:** Univariate Cox analysis of prognostic factors for NPC patients after PSM.

Variables	OS		PFS		DMFS		LRRFS	
	HR (95% CI)	*P*	HR (95% CI)	*P*	HR (95% CI)	*P*	HR (95% CI)	*P*
Gender								
Male vs. female	0.32 (0.11–0.89)	0.030	0.28 (0.11–0.69)	0.006	0.33 (0.12–0.92)	0.034	0.03 (0.00–4.97)	0.178
Age at diagnosis (years)								
18–44	Reference		Reference		Reference		Reference	
45–60	1.38 (0.69–2.77)	0.365	1.77 (0.99–3.19)	0.056	1.92 (0.95–3.86)	0.067	1.67 (0.53–5.27)	0.379
>60	4.71 (1.70–13.05)	0.003	4.09 (1.63–10.27)	0.003	3.83 (1.25–11.78)	0.019	2.37 (0.28–20.41)	0.432
KPS								
≥90 vs. <90	0.95 (0.29–3.08)	0.925	1.35 (0.54–3.39)	0.526	1.55 (0.55–4.37)	0.409	2.06 (0.46–9.31)	0.346
Histological type (WHO)								
I–II vs. III	0.84 (0.38–1.83)	0.656	0.98 (0.49–1.94)	0.942	1.02 (0.45–2.31)	0.972	0.76 (0.21–2.76)	0.675
T category								
T1–2 vs. T3–4	0.77 (0.39–1.51)	0.446	1.05 (0.58–1.91)	0.873	1.16 (0.56–2.40)	0.689	1.38 (0.38–5.03)	0.622
N category								
N0–1 vs. N2–3	1.77 (0.89–3.52)	0.106	1.58 (0.89–2.80)	0.118	1.51 (0.77–2.96)	0.235	1.89 (0.58–6.15)	0.289
Stage								
II–III vs. IVA	2.35 (1.22–4.53)	0.011	2.80 (1.59–4.93)	<0.001	2.57 (1.32–4.99)	0.006	3.57 (1.1–11.62)	0.034
HGB, g/L (M/F)								
≥120/110 vs. <120/110	0.38 (0.05–2.76)	0.338	0.26 (0.04–1.90)	0.186	0.05 (0.00–13.76)	0.288	1.15 (0.15–8.84)	0.895
PLT								
≤300 vs. >300	0.44 (0.21–0.93)	0.031	0.47 (0.25–0.88)	0.018	0.40 (0.19–0.89)	0.023	0.25 (0.05–1.11)	0.068
ALB, g/L								
≥35 vs. <35	1.22 (0.17–8.91)	0.844	0.86 (0.12–6.21)	0.879	NA	NA	NA	NA
IC/AC cycles								
1 vs. ≥2	1.18 (0.52–2.68)	0.696	1.58 (0.75–3.36)	0.232	1.79 (0.70–4.61)	0.224	1.53 (0.34–6.91)	0.580
CC cycles								
1–2 vs. ≥3	1.40 (0.66–2.96)	0.383	1.10 (0.60–2.01)	0.752	0.91 (0.50–1.82)	0.797	1.52 (0.42–5.53)	0.524
Treatment method								
IC + CCRT vs. CCRT + AC	1.02 (0.53–1.96)	0.962	1.31 (0.76–2.26)	0.332	1.62 (0.85–3.09)	0.143	0.93 (0.30–2.83)	0.894

PSM, propensity score matching; IC, induction chemotherapy; AC, adjuvant chemotherapy; KPS, Karnofsky scores; WHO, World Health Organization; HGB, hemoglobin; M/F, male/female; PLT, platelet; ALB, serum albumin; LDH, serum lactate dehydrogenase; alP, serum alkaline phosphatase; sf, serum ferritin; CC, concurrent chemotherapy; CCRT, concurrent chemoradiotherapy; OS, overall survival; PFS, progression-free survival; DMFS, distant metastasis-free survival; LRRFS, locoregional relapse-free survival; HR, hazard ratio; CI, confidence interval; NA, not applicable.

**Table 3:** Multivariate cox analysis of prognostic factors for NPC patients after PSM.

Endpoints	Variables	HR (95% CI)	*P*
**OS**	Gender (male vs. female)	0.33 (0.12–0.94)	0.038
	Age at diagnosis (years) (18–44 vs. >60)	4.50 (1.57–12.89)	0.005
	Stage (II–III vs. IVA)	2.41 (1.23–4.67)	0.009
	PLT (≤300 vs. >300)	0.52 (0.24–1.13)	0.099
	Treatment method (IC + CCRT vs. CCRT + AC)	0.94 (0.49–1.83)	0.865
**PFS**	Gender (male vs. female)	0.28 (0.11–0.71)	0.007
	Age at diagnosis (years) (18–44 vs. >60)	3.98 (1.55–10.18)	0.004
	Stage (II–III vs. IVA)	2.77 (1.57–4.91)	<0.001
	PLT (≤300 vs. >300)	0.59 (0.31–1.12)	0.106
	Treatment method (IC + CCRT vs. CCRT + AC)	1.20 (0.69–2.09)	0.524
**DMFS**	Gender (male vs. female)	0.34 (0.12–0.97)	0.044
	Age at diagnosis (years) (18–44 vs. >60)	3.51 (1.12–11.00)	0.031
	Stage (II–III vs. IVA)	2.49 (1.27–4.88)	0.008
	PLT (≤300 vs. >300)	0.52 (0.23–1.16)	0.108
	Treatment method (IC + CCRT vs. CCRT + AC)	1.46 (0.76–2.80)	0.260
**LRRFS**	Stage (II–III vs. IVA)	3.59 (1.10–11.72)	0.034
	Treatment method (IC + CCRT vs. CCRT + AC)	1.04 (0.34–3.21)	0.940

PSM, propensity score matching; NPC, nasopharyngeal carcinoma; IC, induction chemotherapy; AC, adjuvant chemotherapy; PLT, platelet; CC, concurrent chemotherapy; CCRT, concurrent chemoradiotherapy; OS, overall survival; PFS, progression-free survival; DMFS, distant metastasis-free survival; LRRFS, locoregional relapse-free survival; HR, hazard ratio; CI, confidence interval.

#### 3.1.4 Safety and Toxicity

Safety was summarized by the number of patients experiencing any adverse events, and these data were systematically evaluated and collected using CTCAE 4.03 and RTOG radiation morbidity scoring criteria. The most common acute complications included both hematologic and non-hematologic adverse events (**Table 4**).

**Table 4:** Treatment-related acute toxicities in NPC patients treated with IC + CCRT vs. CCRT + AC after PSM.

Acute toxicities	IC + CCRT		CCRT + AC		*P*-value^a^
	Grades 1–2	Grades 3–4	Grades 1–2	Grades 3–4	
**Hematologic**					
Leukopenia	63 (62.4%)	37 (36.6%)	31 (46.3%)	36 (53.7%)	0.029
Neutropenia	58 (57.4%)	32 (31.7%)	31 (46.3%)	30 (44.8%)	0.085
Thrombocytopenia	23 (22.8%)	5 (5.0%)	8 (11.9%)	3 (4.5%)	1.000
Anemia	95 (94.1%)	1 (1.0%)	61 (91.0%)	1 (1.5%)	1.000
**Non-hematologic**					
Liver dysfunction	45 (44.6%)	6 (5.9%)	27 (40.3%)	0	0.082
Diarrhea	14 (13.9%)	3 (3.0%)	7 (10.4%)	1 (1.5%)	0.922
Nausea/vomiting	80 (79.2%)	11 (10.9%)	58 (86.6%)	1 (1.5%)	0.044
Xerostomia	53 (52.5%)	0	42 (62.7%)	0	–
Mucositis	69 (68.3%)	16 (15.8%)	43 (64.2%)	12 (17.9%)	0.725
Radiodermatitis	67 (66.3%)	3 (3.0%)	49 (73.1%)	2 (3.0%)	1.000
Otitis media	3 (3.0%)	0	1 (1.5%)	0	–

PSM, propensity score matching; NPC, nasopharyngeal carcinoma; IC, induction chemotherapy; CCRT, concurrent chemoradiotherapy; AC, adjuvant chemotherapy.

a For comparison of the difference in the incidence of grade 3–4 toxicities between the treatment groups.

For patients treated with IC + CCRT, the incidence of acute grade 3–4 leukopenia was 37 (36.6%), while 36 (53.7%) was during CCRT + AC. The difference between the two groups was statistically significant (*P* = 0.029). Grade 3–4 nausea/vomiting is more likely to occur during IC + CCRT than during CCRT + AC (10.9% *vs*. 1.5%, *P* = 0.044). There was no significant difference in terms of other toxicities between the two groups.

### 3.2 Meta-Analysis

**Figure 3:**
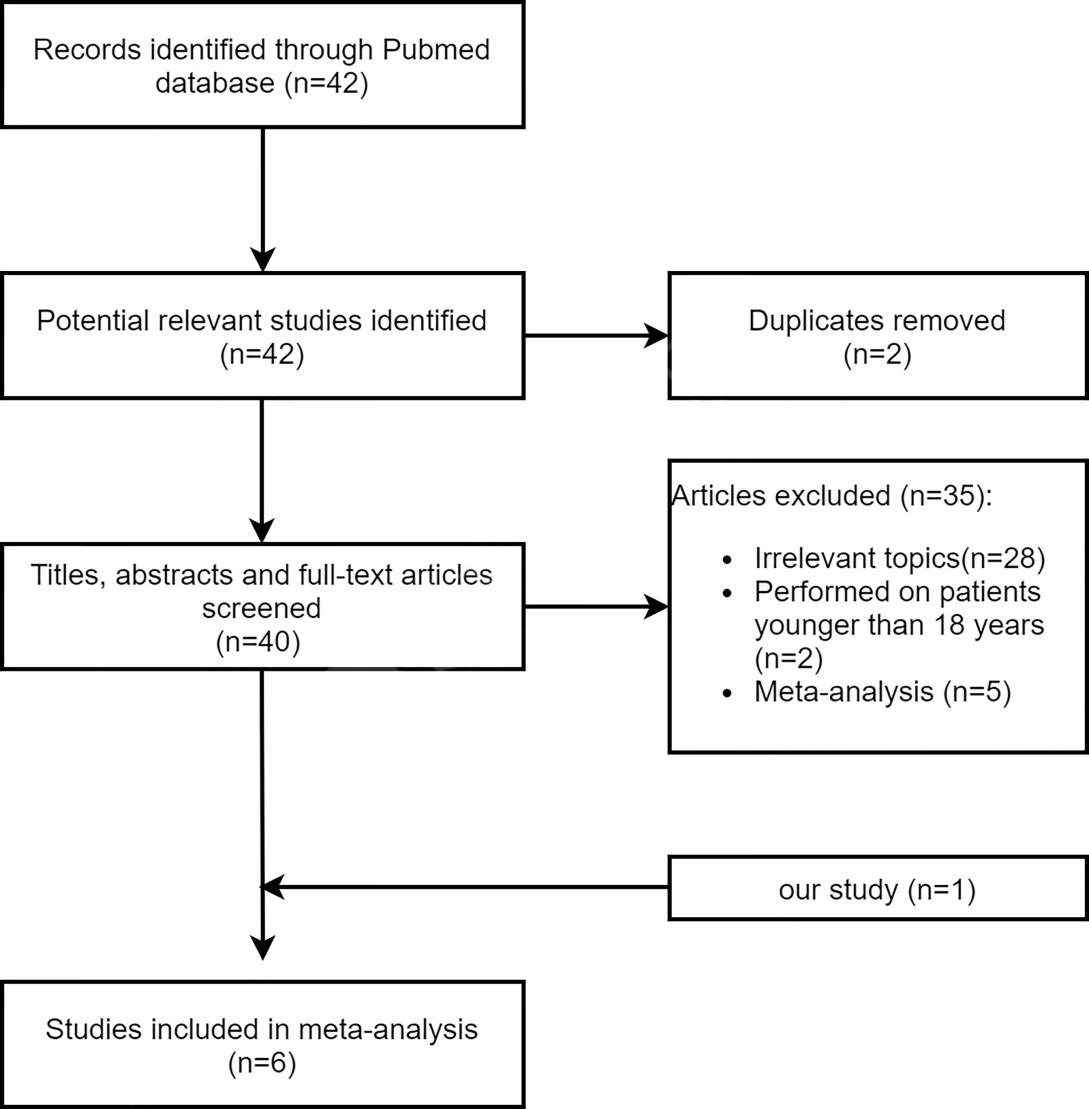
Flowchart illustrating the study selection.

The flowchart of the study selection is shown in **Figure 3**. A total of six articles (including our study) reporting 1,052 cases of IC + CCRT and 883 cases of CCRT + AC in the treatment of NPC were included in our meta-analysis, and their main characteristics are shown in **Table S1** in the **Supplementary Material**. Of the 42 identified articles, 28 articles did not examine the efficacy and/or safety of IC + CCRT and CCRT + AC, 2 articles were repeated, 2 articles were performed on patients younger than 18 years of age, and 5 articles were meta-analysis studies. Consequently, five retrospective studies plus our study were included in the present meta-analysis.

**Figure 4:**
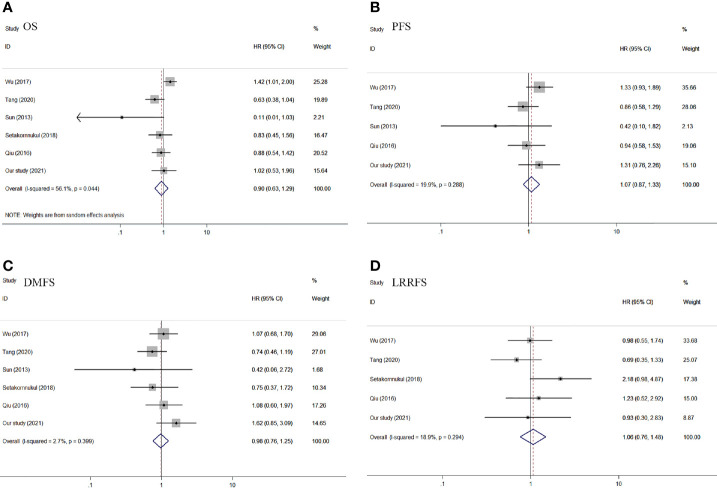
Forest plot of OS **(A)**, PFS **(B)**, DMFS **(C)**, and LRRFS **(D)** of the included studies. OS, overall survival; PFS, progression-free survival; DMFS, distant metastasis-free survival; LRRFS, locoregional relapse-free survival.

For the outcome of OS, data were extracted from six studies (9–13) with 1,935 patients. We used a random-effects model because of the significant heterogeneity (*I* ^2^ = 56.1%, *P* = 0.044) in the included studies. There was no significant difference in OS between IC + CCRT and CCRT + AC (pooled HR = 0.90, 95% CI: 0.63–1.29, *P* = 0.561, **Figure 4A**). Regarding PFS, five articles (9, 11–13) were included in the meta-analysis, which included a total of 1,669 patients. PFS was similar in the IC + CCRT and CCRT + AC (pooled HR = 1.07, 95% CI: 0.87–1.33, *P* = 0.633, **Figure 4B**). On account of no significant difference in the heterogeneity test (*I* ^2^ = 19.9%, *P* = 0.288), a fixed-effect model was applied. Six articles (9–13) reported DMFS with 1,935 patients. There was no significant difference between the two groups in DMFS of the pooled data, with the HR of 0.98 (95% CI: 0.76–1.25, *P* = 0.861, **Figure 4C**). Since the heterogeneity test among the included studies was not statistically significant (*I* ^2^ = 2.7%, *P* = 0.399), we used a fixed-effect model for analysis. For the outcome of LRRFS, five studies (9, 10, 12, 13) with 1,842 patients were appropriate for analysis. There was no significant heterogeneity (*I* ^2^ = 18.9%, *P* = 0.294). In the end, we used a fixed-effect model to calculate pooled data. The results of LRRFS confirmed no significant difference between the IC + CCRT and the IC + RT (pooled HR = 1.06, 95% CI: 0.76–1.48, *P* = 0.724, **Figure 4D**).

**Figure 5:**
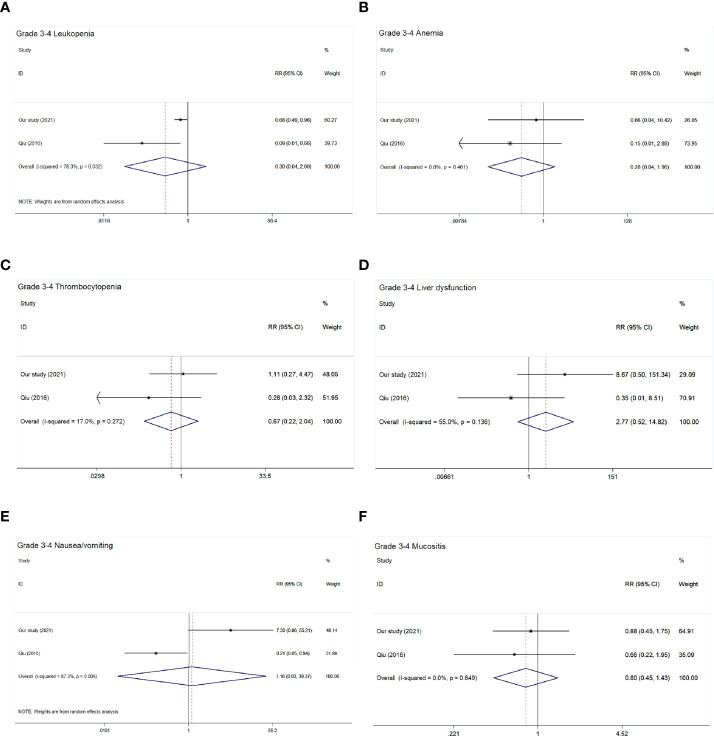
Forest plot of grade 3–4 acute toxic effects of the included studies. **(A)** Grade 3–4 leucopenia; **(B)** Grade 3-4 anemia; **(C)** Grade 3–4 thrombocytopenia; **(D)** Grade 3–4 liver dysfunction; **(E)** Grade 3–4 nausea/vomiting; **(F)** Grade 3–4 mucositis.

Only two studies (9) (including our study) reported adverse reactions. The main acute toxicity forms during treatment include hematologic and non-hematologic adverse events. As shown in **Table 5** and **Figure 5**, there were no significant differences in the incidence of hematologic adverse events such as leucopenia (pooled RR = 0.30, 95% CI: 0.04–2.60, *P* = 0.276), anemia (pooled RR = 0.28, 95% CI: 0.04–1.96, *P* = 0.201), and thrombocytopenia (pooled RR = 0.67, 95% CI: 0.22–2.04, *P* = 0.479) and non-hematologic adverse events such as liver dysfunction (pooled RR = 2.77, 95% CI: 0.52–14.82, *P* = 0.234), mucositis (pooled RR = 0.80, 95% CI: 0.45–1.43, *P* = 0.461), and nausea/vomiting (pooled RR = 1.16, 95% CI: 0.03–39.37, *P* = 0.934).

**Table 5:** Severe toxicity during the IC + CCRT and CCRT + AC in meta-analysis.

Adverse event (grade ≥ 3)	Availability			Effect		Heterogeneity		Analysis model
	Trials (*N*)	IC + CCRT (events/total)	CCRT + AC (events/total)	RR (95% CI)	*P*-value	*I* ^2^	*P*-value	
Leukopenia	2	38/218	48/190	0.30 (0.04–2.60)	0.276	78.3%	0.032	Random effect
Anemia	2	1/218	4/190	0.28 (0.04–1.96)	0.201	0.0%	0.461	Fixed effect
Thrombocytopenia	2	6/218	7/190	0.67 (0.22–2.04)	0.479	17.0%	0.272	Fixed effect
Nausea/vomiting	2	13/218	11/190	1.16 (0.03–39.37)	0.934	87.3%	0.005	Random effect
Mucositis	2	21/218	20/190	0.80 (0.45–1.43)	0.461	0.0%	0.649	Fixed effect
Liver dysfunction	2	6/218	1/190	2.77 (0.52–14.82)	0.234	55.0%	0.136	Fixed effect

IC, induction chemotherapy; AC, adjuvant chemotherapy; CCRT, concurrent chemoradiotherapy; RR, risk ratio; CI, confidence interval.

## 4 Discussion

In this study, a retrospective PSM and meta-analysis showed that IC + CCRT and CCRT + AC had similar survival in stage II–IVA NPC.

Radiotherapy is the main approach for patients with NPC. The addition of chemotherapy, such as IC or AC, based on radiotherapy can reduce treatment failure due to distant metastases (7). Especially for patients with LA-NPC, chemotherapy is unquestionable, and particularly, the combination of concurrent chemotherapy during radiotherapy is of obvious benefit to patients (16). The Intergroup 0099 Study confirmed the survival benefit of concurrent AC in patients with NPC. However, there are still many disputes about its application in popular areas. Then, a study (17) further conducted research in endemic areas, and the results confirmed that the research results of the Intergroup 0099 Study can also be applied in endemic areas of NPC. A phase III clinical trial concluded that adding three cycles of AC to CCRT could not significantly improve the survival rate of LA-NPC compared with CCRT alone. However, in a follow-up study (18), it was considered that AC did not increase late toxicity. The efficacy of IC + CCRT in NPC remains controversial (19, 20). Compared with AC, IC has advantages (16, 21, 22) in improving patient tolerance, reducing the tumor burden, eradicating micrometastasis in the early stage, and reducing tumor to achieve the limit of normal organs when outlining the target area of radiotherapy, which is beneficial to patients with LA-NPC. Hence, IC + CCRT plays an important role in the treatment of patients with LA-NPC in the future. Since then, accumulating evidence has revealed that patients with locoregionally advanced NPC can benefit from IC, and a large number of studies (23–26) have confirmed that the survival benefit of IC + CCRT is more obvious than CCRT alone, and the toxicities can be tolerated. The international guidelines (22) summarized the recommended sequence of chemotherapy regimens to be added to radiotherapy in patients with stage II–IVA NPC. The guidelines concluded that for patients with stage III–IVA (except T3N0) NPC, IC should be administered in addition to CCRT; for patients with stage III–IVA (except T3N0) NPC who do not receive IC + CCRT, CCRT + AC should be administered. However, it is still controversial as to which of the CCRT—combined with IC or AC—has better efficacy, and there is a lack of head-to-head comparative studies of IC + CCRT and CCRT + AC; therefore, it is uncertain which regimen has better efficacy. Currently, clinical studies between IC + CCRT *versus* CCRT + AC in patients with NPC are ongoing (NCT03306121, NCT04898374), and we are looking forward to these results.

In our retrospective study and meta-analysis, IC + CCRT and CCRT + AC showed no significant differences in OS, PFS, DMFS, and LRRFS after PSM. This result is consistent with the results of some of the articles (9, 10) in our included meta-analysis. Nevertheless, in multivariate analysis, our results also suggested that clinical stage (II–III *vs*. IVA) was an independent prognostic factor for OS, PFS, DMFS, and LRRFS. As we all know, stage IVA NPC is defined clinically as T4 or N3 disease without distant metastasis in the UICC/AJCC eighth edition. It has been reported (27) that distant metastasis was the most common failure mode of N3 disease. Other studies (11, 12) have concluded that IC + CCRT may be a reasonable treatment strategy, and this study demonstrated that IC + CCRT leads to a survival advantage over CCRT +AC in T3 or N2 disease. AC is superior to IC in improving LRRFS in T4 disease with no other survival benefit. This is similar to the results of some meta-analysis. A meta-analysis (28) demonstrated that IC + CCRT is the most effective scheme in OS, PFS, and DMFS in the IMRT period compared with CCRT + AC and CCRT. Moreover, compared with CCRT, CCRT + AC achieved the highest survival benefit in terms of LRRFS. In addition, there was a meta-analysis (29) that concluded that the addition of IC to CCRT significantly prolonged OS (HR = 0.64, 95% CI: 0.49–0.84, *P* = 0.001) and PFS (HR = 0.68, 95% CI: 0.56–0.81, *P* < 0.001) in patients compared with CCRT with/without AC. However, there was a corresponding increased risk of grade 3–4 anemia, thrombocytopenia, leukopenia, and fatigue.

Based on our results, after the PSM balanced the variables between the two groups, there was no difference in survival between the two treatment groups. Considering that the survival benefit between the two groups was mainly derived from CCRT, CCRT may have controlled the disease better and reduced the need for chemotherapy in both treatment groups. Regarding the choice of treatment options, clinical decisions should be made holistically based on the individual situation of each patient.

In our study, toxicity was generally manageable in IC + CCRT and CCRT + AC. There were no toxic deaths throughout the study. Grade 3–4 nausea/vomiting is 10.9% during IC + CCRT, which is much higher than CCRT + AC. There may be a potential explanation for this difference. There were 84 (83.2%) patients who completed ≥2 cycles of IC and 50 (74.6%) patients who completed ≥2 cycles of AC. However, IC and AC regimens were based on platinum. Cisplatin is a highly emetogenic chemotherapy drug; therefore, grade 3–4 nausea/vomiting would be heavier than CCRT + AC. On the contrary, the incidence of leucopenia in grades 3–4 was 37 (36.6%) in IC + CCRT and 36 (53.7%) in CCRT +AC, and the difference between them was statistically significant (*P* = 0.029). A possible reason might explain this difference. For patients who have undergone CCRT, the organ of the patient would be more damaged due to the acute effects of CCRT, patients recovering from the toxic effects of CCRT needed more time, and the probability of myelosuppression after AC would be higher than that of IC + CCRT. As a result, the compliance would also be poor, which is similar to some studies (30).

Our study used PSM to balance variables, and thus, it interferes with survival outcomes. Therefore, our research is rigorous. In addition, as far as we know, this is the first meta-analysis using our own research combined with studies published by other centers. However, there are still some limitations in our retrospective study. Firstly, this is a retrospective study. Most patients in our center were treated with IC + CCRT, and the number of cases of CCRT + AC was small. The treatment methods between the two groups were relatively unbalanced, and the sample size was further reduced after PSM. Second, in the CCRT + AC treatment group, due to the large adverse reactions of patients after CCRT treatment, the compliance of adjuvant chemotherapy is worse than that of IC + CCRT. Therefore, patients who required sufficient and appropriate cycles of AC did not return to the hospital on schedule. In addition, the diversity of chemotherapy regimens in our study also influenced our results. There are some limitations in this study. In the first place, because different radiation and chemotherapy treatment records were utilized, these would have an impact on the treatment outcome; except for the data from our center, the included studies were published in the past, and all of them were retrospective studies, which may have selection bias. Secondly, because some HR and CI values could not be provided directly or calculated from the data in some studies, we need to extract the data from the Kaplan–Meier curve, so the values may not be fully accurate, and as the full text of the included articles does not clearly report the values, there may be some marginal errors. There are subjective differences, which will also lead to bias in survival analysis. We look forward to large-scale, prospective clinical trials to further compare the efficacy and safety of IC + CCRT *versus* CCRT + AC.

In conclusion, there are no significant differences in OS, PFS, DMFS, and LRRFS between IC + CCRT and CCRT + AC in stage II-IVA NPC patients. CCRT + AC had a higher incidence of hematologic toxicity than IC + CCRT, and CCRT + AC has a worse compliance than IC + CCRT. IC + CCRT may become the standard treatment for NPC in the future, and we look forward to a long-term, prospective, high-quality clinical study to explore the outcomes and safety of these two treatment patterns.

## Data Availability Statement

The original contributions presented in the study are included in the article/**Supplementary Material**. Further inquiries can be directed to the corresponding author.

## Ethics Statement

The studies involving human participants were reviewed and approved by the Ethics Committee of Guangxi Medical University Cancer Hospital. The ethics committee waived the requirement of written informed consent for participation. Written informed consent was not obtained from the individual(s) for the publication of any potentially identifiable images or data included in this article.

## Author Contributions

Study concepts: X-DZ and JY. Study design: X-DZ, JY, and Z-GL. Data acquisition: JY, Z-GL, Y-TJ, and K-HC. Quality control of data and algorithms: JY and Z-GL. Data analysis and interpretation: JY, Z-GL, Y-TJ, and K-HC. Statistical analysis: JY, Z-GL, SQ, and LL. Manuscript preparation: JY, Z-GL, Y-TJ, and K-HC. Manuscript editing: JY, Z-GL, SQ, and LL. Manuscript review: SQ, LL, and X-DZ. All authors read and X-DZ approved the final manuscript. All authors contributed to the article and approved the submitted version.

## Funding

This research was funded with grants from the National Natural Science Foundation of China (81760544), Guangxi Key Research and Development Program (No. AB18221007), Guangxi Natural Science Foundation Project (2016GXNSFAA380127), Guangxi Medical and Health Appropriate Technology Development and Application Project (S2018001), Guangxi Natural Science Foundation (No. 2020GXNSFBA159002), and the Health Commission of Guangxi Zhuang Autonomous Region (No. Z20200333).

## Conflict of Interest

The authors declare that the research was conducted in the absence of any commercial or financial relationships that could be construed as a potential conflict of interest.

## Publisher’s Note

All claims expressed in this article are solely those of the authors and do not necessarily represent those of their affiliated organizations, or those of the publisher, the editors and the reviewers. Any product that may be evaluated in this article, or claim that may be made by its manufacturer, is not guaranteed or endorsed by the publisher.
