# Supplementary material for: Efficacy and Safety of Concurrent Chemoradiotherapy Combined With Induction Chemotherapy or Adjuvant Chemotherapy in Patients With Stage II–IVA Nasopharyngeal Carcinoma: A Propensity Score Matching Analysis and Meta-Analysis
Source: Front Oncol. 2021 Dec 3;11:778836. doi: 10.3389/fonc.2021.778836 (PMC8678128; doi:10.3389/fonc.2021.778836)
Supplement: Supplementary file 1 [file Table_1.docx]

**Table S1** Main characteristics of all the included studies

| Study | Country | Design type | No. of Patients | | Inclusion period | Median follow-up time, month  (range) | Stage | IC | CCRT | AC | NOS score |
| --- | --- | --- | --- | --- | --- | --- | --- | --- | --- | --- | --- |
|  |  |  |  |  |  |  |  |  |  |  |  |
| Wu, 2017 | China | Retrospective | 618 | 2004.7-2014.12 | | 45.2(1.07-145.4) | Ⅲ-ⅣA (expect T3N0) (8th AJCC/UICC) | PF, TP or TPF | PGTVnx: 66-75 Gy/31-35f, PGTVnd: 65-75 Gy/32-35 f, PCTV1: 56-60Gy/30f, PCTV2: 50Gy/30f, TP or PF as CC regimens | PF, TP or TPF | 6 |
| Tang, 2020 | China | Retrospective | 550 | 2009.4 -2015.12 | | IC+CCRT:49.5  CCRT+AC:55 | Ⅱ-Ⅳ (8th AJCC/UICC) | PF, TP or TPF | PGTVnx: 66-72Gy, PGTVnd: 64-70 Gy, PCTV1: 60-63Gy, PCTV2: 54-56Gy,28-33f，DDP as CC regimens | PF or DDP | 6 |
| Sun, 2013 | China | Retrospective | 93 | 2001.2-2008.1 | | 56(3-120) | Ⅲ-Ⅳ (6th AJCC/UICC) | TC or PF | PGTVnx: 68Gy, PGTVnd: 60-66 Gy, PCTV1: 60Gy, PCTV2: 54Gy,30f，DDP or PF as CC regimens | PF | 6 |
| Setakornnukul, 2018 | Thailand | Retrospective | 266 | 2010.3-2014.10 | | 37 | Ⅱ-ⅣB (7th AJCC/UICC) | PF | PGTVnx and PGTVnd: 70Gy, PCTV1: 60-63Gy, PCTV2: 54-57Gy，DDP as CC regimen | PF | 6 |
| Qiu, 2016 | China | Retrospective | 240 | 2004.1-2008.12 | | NA | Ⅲ-ⅣB (6th UICC) | PF | PGTVnx: 68-70Gy, PGTVnd: 64-68 Gy, PCTV1: 60Gy, PCTV2: 54Gy,30f，DDP as CC regimen | PF | 6 |
| Our study | China | Retrospective | 168 | 2011.11-2015.12 | | 65.2 (7.3-100.9) | Ⅱ-ⅣA (8th AJCC/UICC) | TPF, PF, TP or GP | PGTVnx: 70.06-73.92Gy, PGTVnd: 65.10-72.32Gy, PCTV1: 60-62Gy, PCTV2: 54-55.8Gy, DDP as CC regimen | TPF, PF or TP | 6 |

**Abbreviations:** IC, induction chemotherapy; CCRT, concurrent chemoradiotherapy; AC, adjuvant chemotherapy; CC, concurrent chemotherapy; NOS, Newcastle Ottawa Scale; AJCC, American Joint Committee on Cancer; UICC, Union for International Cancer Control; PF, cisplatin and 5-fluorouracil; TPF, docetaxel combined with cisplatin and fluorouracil; TP, docetaxel and cisplatin; DDP, cisplatin; GP, gemcitabine and cisplatin; TC, paclitaxel and carboplatin; GTV, gross tumor volume; CTV, clinical target volume; PTV, planning target volume; NA, not available.
